# Supplementary material for: From sequence to enzyme mechanism using multi-label machine learning
Source: BMC Bioinformatics. 2014 May 19;15:150. doi: 10.1186/1471-2105-15-150 (PMC4229970; doi:10.1186/1471-2105-15-150)
Supplement: Additional file 2 — Java code of ml2db. Additional file ml2db_code.tar.gz contains the Java source code to run the multi-label machine learning experiments and save the results to database. The code’s Javadoc is included. [file 1471-2105-15-150-S2.zip › additional file 2/ml2db/utils/doc/uk/ac/ed/inf/utils/EntrezUtils.html]

EntrezUtils


---


|  |  |  |
| --- | --- | --- |
| **Overview** \| **Package** \| **Class** \| **Use** \| **Tree** \| **Deprecated** \| **Index** \| **Help** | |  |
| PREV CLASS   **NEXT CLASS** | **FRAMES**    **NO FRAMES**     **All Classes** |  |
| SUMMARY: NESTED \| FIELD \| CONSTR \| METHOD | DETAIL: FIELD \| CONSTR \| METHOD |  |


---


## uk.ac.ed.inf.utils Class EntrezUtils

```
java.lang.Object
  uk.ac.ed.inf.utils.EntrezUtils
```

---

``` public class EntrezUtils extends java.lang.Object ```

Utilities for Entrez services.

**Version:**
:   12 Dec 2007

**Author:**
:   Luna De Ferrari luna.deferrari-at-ed.ac.uk

---

**Constructor Summary**

`EntrezUtils()`


| **Method Summary** | |
| --- | --- |
| `static java.lang.String` | `eSummaryToString(XmlNode xmlTree)`             Test method to print an an Entrez eSummary xml report |
| `static XmlNode` | `getESummaryXML(java.lang.String db, java.lang.String id)`             Gets an XML tree of elements (SimpleElement) from eSummary Entrez service. |
| `static java.lang.String` | `getNameForEntrezGeneId(java.lang.String id)`             Get the entrez gene name for an entrez gene id |
| `static java.lang.String` | `getPubMedUrl(java.lang.String pubMedIds)`             Generates pubMed URL adding pubMed ids (as list separated by commas) |
| `static void` | `main(java.lang.String[] args)`             Mains for testing |
| `static java.lang.String` | `xmlElementToString(XmlNode xml)`             Test method to print to string a single Entrez eSummary xml element |

**Methods inherited from class java.lang.Object**

`equals, getClass, hashCode, notify, notifyAll, toString, wait, wait, wait`

**Constructor Detail**

### EntrezUtils

```
public EntrezUtils()
```


**Method Detail**

### eSummaryToString

```
public static java.lang.String eSummaryToString(XmlNode xmlTree)
```

:   Test method to print an an Entrez eSummary xml report

    :   **Parameters:**: `xmlTree` - **Returns:**: to string

---


### getESummaryXML

```
public static XmlNode getESummaryXML(java.lang.String db,
                                     java.lang.String id)
                              throws java.io.IOException
```

:   Gets an XML tree of elements (SimpleElement) from eSummary Entrez
    service. Example: //
    http://eutils.ncbi.nlm.nih.gov/entrez/eutils/esummary
    .fcgi?db=gene&id=55111&retmode=xml

    :   **Parameters:**: `db` - a database (for example for Entrez gene = 'gene'): `id` - if database = gene > an entrez gene id **Returns:**: an xml simple element **Throws:**: `java.io.IOException` **See Also:**: `XmlNode`

---


### getNameForEntrezGeneId

```
public static java.lang.String getNameForEntrezGeneId(java.lang.String id)
                                               throws java.io.IOException
```

:   Get the entrez gene name for an entrez gene id

    :   **Parameters:**: `id` - the entrez gene id, eg. 1956 (EGFR) **Returns:**: the entrez gene name, eg. 'EGFR' (for entrez gene id 1956) **Throws:**: `java.io.IOException`

---


### getPubMedUrl

```
public static java.lang.String getPubMedUrl(java.lang.String pubMedIds)
```

:   Generates pubMed URL adding pubMed ids (as list separated by commas)

    :   **Parameters:**: `pubMedIds` - eg. 16381840,15608222 **Returns:**: pubMed fetch url, eg: http://www.ncbi.nlm.nih.gov/sites/entrez?cmd =retrieve&db=pubmed&list\_uids=16381840,15608222&dopt=citation

---


### main

```
public static void main(java.lang.String[] args)
                 throws java.io.IOException
```

:   Mains for testing

    :   **Parameters:**: `args` - **Throws:**: `java.io.IOException`

---


### xmlElementToString

```
public static java.lang.String xmlElementToString(XmlNode xml)
```

:   Test method to print to string a single Entrez eSummary xml element

    :   **Parameters:**: `xml` - **Returns:**: the string value of the xml element


---


|  |  |  |
| --- | --- | --- |
| **Overview** \| **Package** \| **Class** \| **Use** \| **Tree** \| **Deprecated** \| **Index** \| **Help** | |  |
| PREV CLASS   **NEXT CLASS** | **FRAMES**    **NO FRAMES**     **All Classes** |  |
| SUMMARY: NESTED \| FIELD \| CONSTR \| METHOD | DETAIL: FIELD \| CONSTR \| METHOD |  |


---
